# Concerning Various Methods Advocated for Obviating the Necessity of Extracting Devitalized Tooth-Pulps

**Published:** 1893-10

**Authors:** W. D. Miller

**Affiliations:** Berlin, Germany


### CONCERNING VARIOUS METHODS ADVOCATED FOR OBVIATING THE NECESSITY OF EXTRACTING DEVITALIZED TOOTH-PULPS.[1]

BY DR. W. D. MILLER, BERLIN, GERMANY.

THE practice now in vogue among good practitioners, of thoroughly removing the pulp and filling the root-canal to the apex, is usually so easily carried out in the incisors and cuspids, and gives such sure results, that there is no probability that a better method will ever be found. But when we extend this treatment to the bicuspids and molars, the labor and expense entailed are frequently so great as to put it beyond the reach of the great majority of the human race, and the method is not always successful. It will conse-

---

[1] Abstract from Dr. Miller's paper read before the World's Columbian Dental Congress, Chicago, August, 1893.

quently be a great boon if some means or method can be devised which would render unnecessary the removing of the pulp and filling the root-canals of molars.

While every dentist has now and then knowingly left remains of the pulp in narrow and tortuous canals, or in canals obstructed by calcific matter, and while many dentists in Europe have contented themselves with simply devitalizing the pulp, filling over it with amalgam and *leaving the rest to nature*, the first *systematic* attempt to do away entirely with the necessity of extracting the root-portions of the pulp appears to have been made by Witzel, who in 1874 presented the view that an application of arsenous acid carefully made to the inflamed pulp devitalized only the diseased tissue, and that by amputating the coronal portion of the pulp twenty-four hours after the application, the ends of the root-stumps might be treated as healthy, freshly-exposed pulps.

Dr. Miller then presented briefly the methods devised by Witzel, Baume, and Herbst, the latter as put forth by its author and as modified by Bödecker, and summarized their advantages and disadvantages. Continuing, he said,—

Perhaps the majority of dentists have also made more or less extensive use of the method recommended by Bödecker when they have left a portion or the whole of the pulp in the buccal roots of upper or the mesial roots of lower molars, and filled directly over them, after thoroughly bathing them with carbolic acid or some other antiseptic.

I have for a long time felt that the solution of the problem was to be sought for in the direction pointed out by Witzel, except that our efforts should be directed not to retaining the vitality of the root-stumps, but to preventing their subsequent decomposition by impregnating them with a suitable antiseptic. I am convinced that the success of the impregnation method depends to a very great extent upon the character of the antiseptic employed, and upon its chemical action upon the pulp apart from its antiseptic action.

The qualities desirable appear to me to be,—

1. It must be a strong antiseptic.

2. It must be sufficiently soluble and diffusible to guarantee the impregnation of the whole pulp.

3. It must not be so diffusible that it will be completely taken up by the surrounding tissue and finally disappear altogether, as is the case with applications of carbolic acid. It is my impression that there is greater danger in too great solubility than in insolubility.

4. A coagulating action upon the tissue of the pulp appears desirable, though not absolutely essential. A pulp which is coagulated into a hard, insoluble body is less likely to furnish nourishment for bacteria and offer irritation to the periapical tissue than one in a soft or semi-liquid condition. One cause of the failure of Baume's borax treatment is probably the conversion of the pulp into a liquid or semi-liquid, soapy mass with a strong alkaline smell and reaction, which can hardly be indifferent to the tissue about the apical foramen.

5. It is desirable that the substance employed have no irritating action upon the pericementum.

6. It should not discolor the tooth, although, as the treatment concerns chiefly molars, a slight discoloration need not be considered as a very serious matter.

7. Solid substances are better adapted to the purpose than liquids.

It is difficult to find a substance which fulfils all the above-mentioned conditions.

According to the results obtained from over five hundred experiments, I have divided dental antiseptics into three groups,—

1. Those possessing in a high degree the power of imparting antiseptic qualities to root-pulps, such as cyanide of mercury, bichloride of mercury, diaphtherin, sulphate of copper, salicylate of mercury, oil of cinnamon, ortho-kresol, carbolic acid, trichlor-phenol, chloride of zinc. The last four are, however, decidedly inferior to the others; they penetrate the pulp very rapidly, chloride of zinc surprisingly so, but they are lacking in the necessary powerful antiseptic qualities, and are so diffusible that in the course of a few weeks they disappear altogether from the pulp.

2. Those of doubtful value: Thymol, salicylic acid, eugenol, campho-phénique, hydronaphthol, A and B naphthol, acetico-tartrate of aluminum, and some essential oils, resorcin, thallin, sulpho-carbolate of zinc, oil of birch, iodide of sodium, nitrate of sodium, etc.

3. Those nearly or quite worthless: Iodoform, basic aniline coloring-matters, borax, boracic acid, dermatol, europhen, chloride of lime, peroxide of hydrogen, sozoiodol salts, iodol, tincture of iodine, spirits of camphor, naphthalin, etc.

The attempt to apply these results to practice was first made with the bichloride of mercury, which has been used since 1890 in some four hundred to five hundred cases, first in the form of small tablets, having the composition,—

Sublimate, 0.01 gramme;
Boracid acid, 0.02 gramme.

Or,

Sublimate, 0.01 gramme;
Common salt, 0.02 gramme.

The pulp having been completely devitalized, the pulp-chamber was thoroughly opened and cleansed, and a tablet applied and slightly crushed with an amalgam plugger, moistened with water and covered with a layer of tin-foil (I now use gold-foil), and the amalgam or cement filling immediately inserted. In about thirty per cent. of the cases severe pain occurred on the day following the application, and on account of this disagreeable symptom these tablets were abandoned and the following substituted:

Sublimate, 0.0075 gramme;
Thymol, 0.0075 gramme.

These are applied in the same manner. The thymol being chiefly designed to prevent the sublimate being so rapidly absorbed, besides giving a greater permanency to the application, by reducing its solubility. Very seldom, so far, has pain followed the use of these tablets, while experiments out of the mouth show that they still possess sufficient penetrating power.

Another combination employed is,—

Sublimate, 0.005 gramme;
Thymol, 0.005 gramme;
Tannin, 0.005 gramme.

This combination is somewhat empirical, though the design of the tannin will be apparent to every one. The combination does not penetrate as rapidly as No. 2, and discolors the tooth more.

Cyanide of mercury has also been employed in combination with thymol, in the following form:

Cyanide of mercury, 0.0075 gramme;
Thymol, 0.0075 gramme.

Also the salicylate of mercury in the same form. This I think deserving of a trial. Its sparing solubility justifies the belief that its action will be more permanent than that of sublimate. The sulphate of copper may be used in pure form, but it naturally causes serious discoloration of the tooth at the neck, and is also, I fear, too soluble to give permanent results, in pure form. More recently, I have directed my experiments towards the discovery of some substance which possesses the desired qualities without discoloring the tooth. Thus far I have obtained the best result from diaphtherin

(oxychinaseptol), an antiseptic recently introduced by Emmerich. It may be applied in pure form. Among liquid antiseptics, the oil of cinnamon takes the first place, and I have much faith in its power to conserve the dead pulp. Like all the liquids, however, it is difficult to apply, and has, besides, the disagreeable quality of discoloring the tooth yellowish-brown. The combination which I have chiefly employed is that of sublimate and thymol. (I have not had opportunity to sufficiently test the others in practice, though I am now using, by way of experiment, the salicylate, and, to some extent, the cyanide of mercury.) It has been employed at the Dental Institute of the University of Berlin in over two hundred cases. Of these, only one failure has come to my knowledge.

Time is the only test for methods like those under consideration, and we can scarcely expect to arrive at a definite conclusion in less than five to ten years. Nor should we be hasty in the application of methods of this nature. One or two cases every month, at least for the first year or two, is all that a careful dentist ought to risk in private practice. Cases should be chosen which are very difficult to treat and which are otherwise frequently treated by the forceps, such as distal cavities of second or third molars, buccal cavities of third molars, etc. It is not possible at present to form a reliable estimate as to the value of this method of treating teeth; it may also be that much better materials will be found for the purpose than those suggested above. There are, at least, reasons for believing that by a careful application of this method many teeth may be saved which otherwise would be sacrificed to the forceps, or, what is much worse, be allowed to crumble away.

[The President exhibited two small bottles containing the preparations which Dr. Miller had recommended, and passed them around for inspection.]

